# Thermodynamic and Structural Evidence for Higher-Order Selegiline with Carboxymethyl-β-Cyclodextrin Supramolecular Assemblies in Aqueous Solution

**DOI:** 10.1021/acs.jpcb.6c03442

**Published:** 2026-09-06

**Authors:** Diego Caproni de Morais, Rogério R. Macedo, Bianca B. M. Vieira, Ivana Lula, Juliana Fedoce Lopes, Frederico B. De Sousa

**Affiliations:** † Laboratório de Sistemas Poliméricos e Supramoleculares (LSPS)–Instituto de Física e Química, Universidade Federal de Itajubá (UNIFEI), Itajubá 37500-903, M G, Brazil; ‡ Laboratório de Química Computacional (LaQC)−Instituto de Física e Química, Universidade Federal de Itajubá (UNIFEI), Itajubá 37500-903, Brazil; § Departamento de Química−Instituto de Ciências Exatas, Universidade Federal de Minas Gerais (UFMG), Belo Horizonte 31270-901, MG, Brazil

## Abstract

The supramolecular interactions between selegiline hydrochloride (SEL) and β-cyclodextrin (βCD) derivatives were investigated through isothermal titration calorimetry (ITC), nuclear magnetic resonance (NMR), and molecular dynamics (MD) simulations. Thermodynamic analyses revealed spontaneous complex formation in aqueous solution for both βCD and carboxymethyl-β-cyclodextrin (CM-βCD), with CM-βCD exhibiting higher equilibrium constants and more favorable thermodynamic parameters. The complexation process was predominantly entropy-driven, indicating significant contributions from hydrophobic interactions and solvent reorganization. Non-integer stoichiometric values (*n* ≈ 1.5) obtained by ITC suggested the formation of higher-order supramolecular assemblies. NMR experiments demonstrated strong spatial correlations between the aromatic hydrogens of SEL and the cavity hydrogens of CM-βCD, supporting deep inclusion within the CD cavity. MD simulations corroborated the experimental findings and demonstrated the feasibility of a 3:2 CM-βCD:SEL supramolecular organization. Overall, the combined thermodynamic, spectroscopic, and computational results provide compelling evidence for higher-order, multi-equilibrium cyclodextrin supramolecular assemblies in aqueous solution.

## Graphical abstract

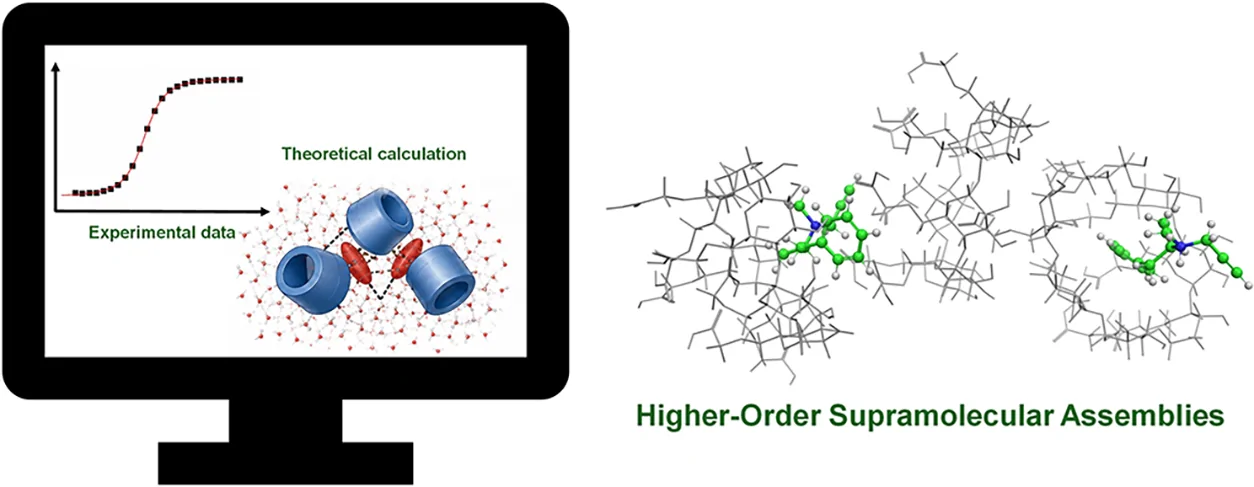

## 1 Introduction

Supramolecular chemistry has emerged as a central field in modern chemical science by focusing on the organization and function of molecular systems formed through non-covalent interactions such as hydrogen bonding, electrostatic forces, van der Waals interactions, and hydrophobic effects.^1,2^ These reversible and highly specific interactions enable the construction of complex, adaptive architectures with applications ranging from drug delivery and molecular recognition to catalysis and smart materials.^3–5^ Within this context, cyclodextrins (CDs) have attracted considerable attention as versatile supramolecular hosts because of their cyclic structure, which provides a hydrophobic internal cavity and a hydrophilic exterior. This unique architecture allows CDs to form host–guest inclusion complexes with a wide variety of molecules.^6^ Consequently, CD-based systems have become key platforms in the design of functional supramolecular assemblies for pharmaceutical, environmental, and materials science applications.^7^

Building upon their role as supramolecular hosts, the formation of inclusion complexes (ICs) between CDs and guest molecules is governed by fundamental physicochemical principles of molecular recognition in solution and the solid state.^8,9^ The truncated cone structure of CDs creates a hydrophobic internal cavity surrounded by a hydrophilic outer surface, which enables the encapsulation of suitably sized guest molecules. Complex formation is mainly driven by the hydrophobic effect, van der Waals interactions, hydrogen bonding, and the displacement of energetically unfavorable water molecules from the CD cavity, resulting in a favorable change in the Gibbs free energy.^10^ From a thermodynamic standpoint, these host–guest systems are described by the equilibrium between free host and guest molecules and their complexes. Although the most common stoichiometry corresponds to a 1:1 host–guest complex, other arrangements such as 2:1 (two CD molecules associated with a single guest) or 1:2 complexes may also occur depending on the size, geometry, and binding sites of the guest molecule.^11,12^ However, in many systems, multiple binding equilibria may coexist simultaneously in solution, leading to more complex association models.^13–15^

To better understand the equilibrium behavior of CD host–guest systems, the characterization of supramolecular species involves both experimental and computational approaches in solution and the solid state.^16,17^ Among the various techniques used to study molecular interactions, isothermal titration calorimetry (ITC) stands out as an effective tool for investigating the complexation between CDs and their guests.^18^ In addition to providing detailed thermodynamic data for the interaction process,^19^ ITC allows the evaluation of the stability of ICs under different experimental conditions, such as pH and temperature variations.^20^ However, despite its versatility, ITC has some limitations, including the relatively long duration of experiments, the requirement for the analytes to exhibit high solubility in aqueous media, and its inability to provide detailed structural information about the resulting supramolecular systems.^21^ Furthermore, although ITC provides valuable thermodynamic information, it does not directly reveal the structural arrangement of the ICs, making complementary techniques such as nuclear magnetic resonance (NMR) spectroscopy and theoretical calculations essential for elucidating the supramolecular organization.^22,23^

In this study, selegiline hydrochloride (SEL) was chosen as a model guest molecule (Figure 1). SEL is a drug widely used for the treatment of Parkinson’s disease, acting as an irreversible and selective inhibitor of monoamine oxidase type B (MAO-B).^24,25^ SEL was selected as a model guest molecule because of its chemical structure, aqueous solubility, and pharmaceutical relevance. At higher doses, SEL can also inhibit monoamine oxidase type A (MAO-A), thereby enhancing its antidepressant potential, which has led to its use in the treatment of depression, particularly in transdermal formulations.^26^

**Figure 1:**
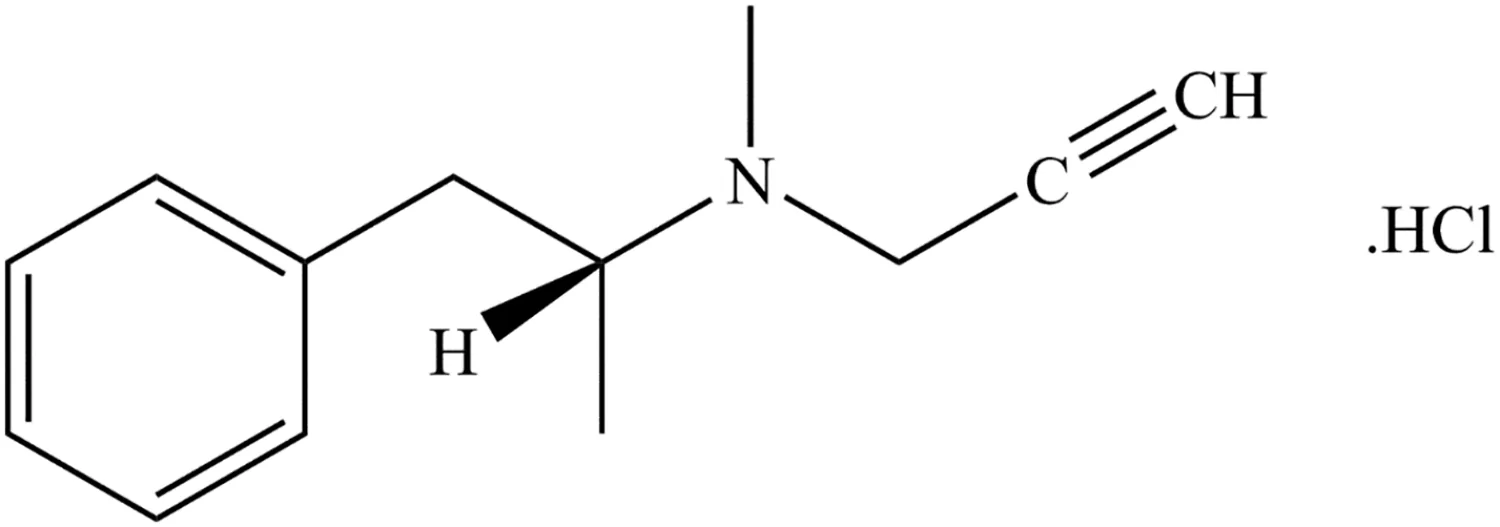
Chemical structure of selegiline hydrochloride.

Despite its therapeutic benefits, the use of SEL is associated with some adverse effects, such as insomnia, nausea, dry mouth, orthostatic hypotension, fainting, agitation, and tachycardia. Additionally, it has low bioavailability (∼10 %), primarily attributed to first-pass metabolism.^27^ Given its applications and pharmacological limitations, SEL represents an attractive molecular model for investigating the thermodynamic and structural features governing CD-mediated host–guest interactions in aqueous solution. Herein, the supramolecular interaction between SEL and natural and modified CDs was investigated using ITC and NMR spectroscopy, combined with molecular dynamics (MD) simulations. Combining these methods not only enabled the characterization of the thermodynamic properties and supramolecular morphology of the systems but also provided evidence for higher-order stoichiometries, whose experimental elucidation remains challenging due to the dynamic coexistence of multiple equilibria in aqueous solution.

## 2 Experimental Section

### 2.1 Reagents and Solvents

The following reagents, αCD (CAS: 10016-20-3, ≥98.0 %), βCD (CAS: 7585-39-9, ≥98.0 %), γCD (CAS: 17465-86-0, 98.3 – 102.0 %), and the sodium salt of carboxymethyl-β-cyclodextrin, CM-βCD (CAS: 2828447-14-7, >95 %), were acquired from Sigma-Aldrich. Selegiline hydrochloride (SEL) was purchased from Araujo S.A. (≥95.0 %). All reagents were used without further purification.

### 2.2 Isothermal Titration Calorimetry

Isothermal titration calorimetry experiments were carried out using a VP-ITC microcalorimeter (Microcal). The thermodynamic parameters were determined using CD solutions in the titration syringe and the guest molecule (SEL) in the reaction cell (the sample concentrations are presented in Table 1). The concentration of each CD used during the titration experiments varied depending on the host molecule in order to improve the measured heat signal and maximize the interaction between the host and guest molecules.^28^ A total of four supramolecular systems were investigated by ITC, using three natural CDs and one modified CD.

**Table 1:** Concentrations Used for the Titration of Aqueous CD Solutions into Aqueous SEL Solution

	αCD	βCD	γCD	CM-βCD
[CD] mmol L^–1^	14.0	14.0	14.0	20.0
[SEL] mmol L^–1^	1.0	1.0	1.0	1.0

All titrations were carried out by performing 25 successive injections of the aqueous CD solution into the aqueous SEL solution. Each titration was performed with an interval of 300 s between consecutive injections, at 298.15 K. In addition, the titration of CM-βCD into aqueous SEL was performed at 298.15, 308.15, 318.15, and 328.15 K to obtain further insights into the thermodynamic parameters governing the supramolecular interaction. For all titrations, the first injection (1.0 μL) was discarded to eliminate the injection-related dispersion effects, and the subsequent injections consisted of 10.0 μL. The calorimeter reference cell was filled with Milli-Q water, and all samples were prepared using Type 1 Milli-Q water (18.2 mΩ·cm @ 25 ^o^C). In addition, control titrations were carried out by injecting CD (natural or modified) aqueous solutions into Milli-Q water and Milli-Q water into aqueous SEL solutions to correct for the heat of dilution and solvent mixing. The calorimetric data were analyzed using Origin 7.0 (MicroCal for ITC). The stoichiometry coefficient (*n*), equilibrium constant (*K*_eq_), and standard enthalpy variation (Δ*H* ^o^) were obtained by nonlinear curve fitting (one-set-of-sites binding model). Standard Gibbs free energy change (Δ*G* ^o^) and standard entropy contribution (*T*Δ*S* ^o^) were calculated using fundamental thermodynamic eqs ([Disp-formula eq1]) and ([Disp-formula eq2]).

1 $$\Delta G^{{}^{\circ}}=-\mathrm{RT}\ln K_{eq}$$

2 $$\Delta G^{{}^{\circ}}=\Delta H^{{}^{\circ}}-T\Delta S^{{}^{\circ}}$$

### 2.3 Nuclear Magnetic Resonance

NMR experiments were carried out using a Bruker ONEBAY ASCEND 400 spectrometer operating at a ^1^H resonance frequency of 400 MHz at 27 °C using 5 mm NMR tubes. SEL (5.0 mg) was dissolved in 0.7 mL of deuterium oxide (D_2_O) containing 5% (v/v) DMSO*d*_6_, and a series of one- and two- dimensional homo- and heteronuclear NMR experiments was conducted to assign the chemical shifts of the drug molecule. In addition, the experiments were performed at 50 ^o^C to improve the spectral resolution. The CM-βCD:SEL IC (10.0 mg, 1:1 molar ratio) was dissolved in 0.7 mL D_2_O. One-dimensional ^1^H NMR and two-dimensional rotating-frame Overhauser effect spectroscopy (^1^H/^1^H ROESY) experiments were performed to confirm the host–guest interactions in solution. In addition, the ^1^H NMR spectrum of CM-βCD (10.0 mg dissolved in 0.7 mL of D_2_O) was acquired (Figure S1), and the observed chemical shifts were in accordance with previously reported values.^29^

### 2.4 Theoretical Calculations

The structure of CM-βCD used in the molecular dynamic (MD) simulation was optimized using Gaussian 09 at the M06-2X functional and the 6-31 + G­(d,p) level of theory. The initial structure of SEL was obtained from the Cambridge Crystallographic Data Centre (CCDC code FADBUH) and was optimized using Gaussian 09 at the B3LYP functional and the 6-31g+ level of theory. Based on the optimized structures (Figure S2 and Table S1), the initial MD structure was built by positioning the molecules with their centers of mass aligned along a common reference axis, followed by manual orientation. Subsequently, the structures were initially separated by 12 Å. MD simulations were performed using the GROMACS 2024 package^30^ and the OPLS-AA^31^ force field. The SPC/E water model was employed.^32^ Parameters for CM-βCD and SEL, compatible with the OPLS-AA force field, were generated using the LigParGen web server.^32^ A cubic simulation box with a dimension of 5 nm and periodic boundary conditions was used. Water molecules were randomly added to fill the simulation box of the box, and the CM-βCD and SEL structures were placed at its center.

The MD simulation was designed to investigate the formation of host–guest ICs between SEL and CM-βCD. The simulation followed four main steps: (1) energy minimization, (2) NVT equilibration, (3) NPT equilibration, and (4) the production run. Energy minimization was performed using the steepest descent method, with a maximum of 100,000 steps. During the NVT equilibration, a reference temperature of 298.15 K was maintained using the velocity-rescale thermostat with a coupling time (tau-t) of 0.1 ps. During the NPT equilibration, a reference pressure of 1 bar was used, with the Parrinello–Rahman barostat and a coupling time (tau-p) of 5.0 ps. During the production run, the parameters from the NVT and NPT equilibration stages were maintained. The NVT and NPT equilibration stages were conducted for 2 ns and 10 ns, respectively, followed by a 100 ns production simulation. Theoretical correlations of the distances among hydrogen atoms to simulate the 2D ^1^H/^1^H ROESY NMR counter maps were obtained using the program, as previously described.^33^

## 3 Results and Discussion

### 3.1 Isothermal Titration Calorimetry: Native CDs

ITC is a powerful analytical technique widely employed to investigate the thermodynamics of molecular interactions in solution. Its significance is particularly pronounced in the study of supramolecular systems involving CDs and guest molecules, where interactions are often weak and driven by subtle enthalpic and/or entropic contributions.^34^ The intermolecular interaction between the SEL drug molecule and the three most commonly used natural CDs (αCD, βCD, and γCD) was first investigated. Figure 2a–c depicts the ITC titration curves obtained for the titration of αCD, βCD, and γCD aqueous solutions into the SEL aqueous solution, respectively, together with their corresponding dilution curves.

**Figure 2:**
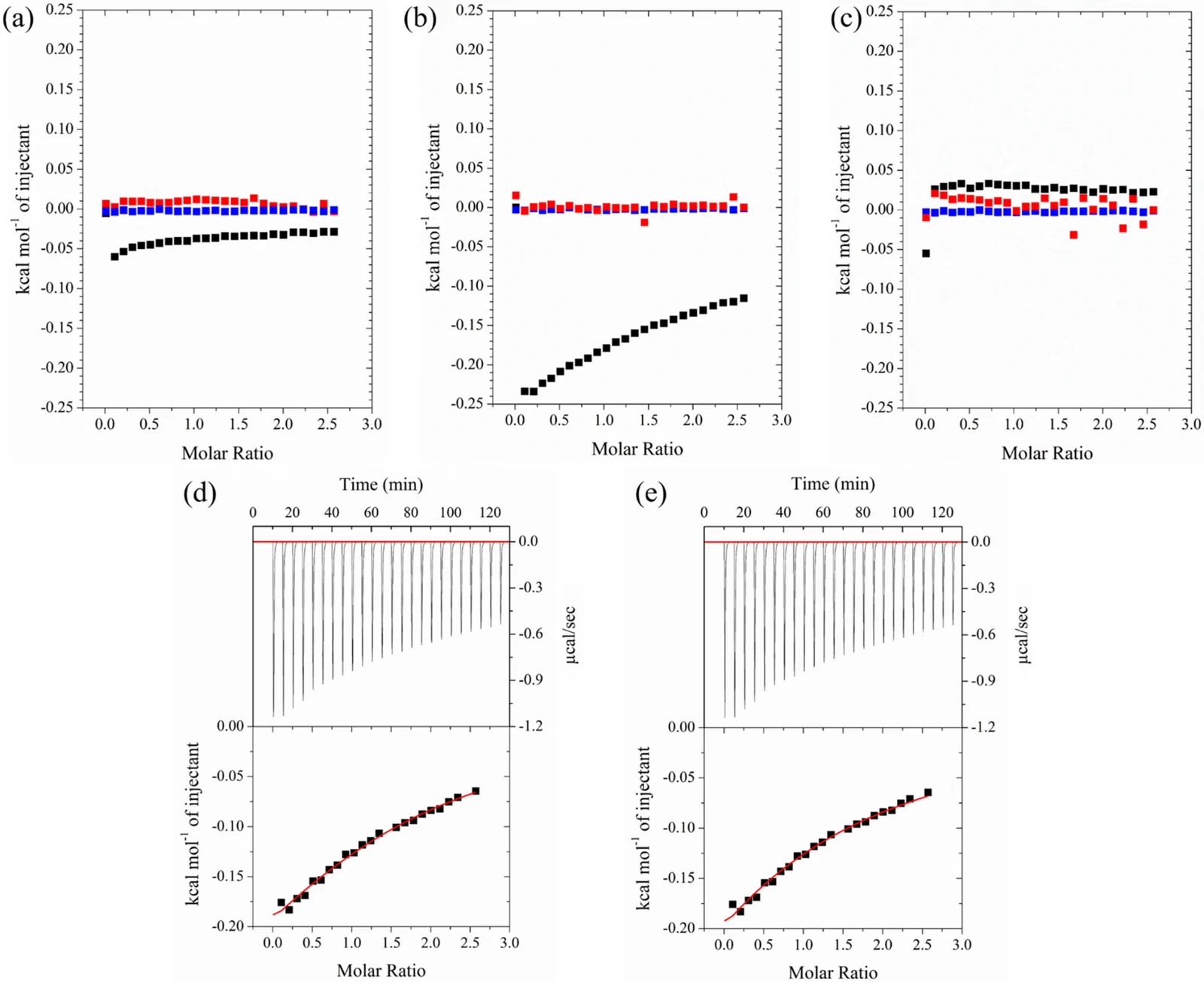
ITC titration curves (at 298.15 K) for the supramolecular systems (■) and host (■) and guest (■) dilution curves for: (a) αCD:SEL, (b) βCD:SEL, and (c) γCD:SEL. ITC final figures for the βCD:SEL titration process with (d) variable stoichiometry and (e) fixed (1:1) stoichiometry.

As shown in Figure 2a,c, the heat exchanged during the titration of αCD and γCD into the SEL aqueous solution is comparable to that observed for the corresponding dilution experiments, demonstrating a very weak binding affinity between these hosts and the guest drug molecule. Consequently, the interaction is too weak to allow reliable determination of the thermodynamic binding parameters. The lower affinities observed for αCD and γCD, compared with the βCD:SEL supramolecular system, can be rationalized by the relationship between the host cavity size and the dimensions of the guest molecule. The cavity of αCD is too small to accommodate the guest drug or its functional moieties, limiting the inclusion process, whereas the cavity of γCD is larger than the guest molecule, reducing the effective intermolecular interaction between them. A similar behavior has been reported for other supramolecular systems.^35^

However, a distinct heat flow was observed during the titration of βCD into the SEL aqueous solution when compared with the corresponding dilution experiment (Figure 2b). The thermodynamic parameters obtained for this supramolecular system are summarized in Table 2. Initially, all fitting parameters were allowed to vary freely during the nonlinear fitting procedure, resulting in a stoichiometric coefficient (*n*) of approximately 1.6. This non-integer stoichiometry suggests the formation of higher-order supramolecular systems in aqueous solution, rather than a simple 1:1 host–guest complex.^15^ Nevertheless, because the molecular dimensions of SEL are consistent with inclusion within a single βCD cavity, an additional fitting was performed by fixing the stoichiometry at a 1:1 host-to-guest molar ratio. This analysis was carried out to enable comparison with the conventional binding model commonly reported for CD Ics. The thermodynamic parameters obtained from both fitting procedures are presented in Table 2. The corresponding fitted ITC curves for the βCD:SEL supramolecular system, using variable (*n* = 1.6) and fixed (*n* = 1.0) stoichiometries, are shown in Figure 2d,e, respectively, demonstrating that both models provide similarly satisfactory descriptions of the experimental data.

**Table 2:** Thermodynamic Parameters Describing the Supramolecular Interaction between βCD and SEL in Aqueous Solution at 298.15 K

CD	*n*	*K*_eq_	Δ*H*° / kJ mol^–1^	*T*Δ*S*° / kJ mol^–1^	Δ*G*° / kJ mol^–1^
βCD	1.63	525	–1.71	13.85	–15.55
βCD	1.00	302	–3.47	10.68	–14.15

Although a reduction in the equilibrium constant (*K*_eq_), from 525 to 302, was observed when the stoichiometry was fixed at 1:1, the magnitude of the values remained comparable. An analogous comparison can be made for the remaining thermodynamic parameters of the βCD:SEL supramolecular system, considering both the fixed 1:1 stoichiometry and the variable stoichiometry model. The interaction between βCD and the SEL drug molecule was found to be spontaneous, based on the Δ*G* ^o^ value (−15.55 kJ mol^–1^ for the variable stoichiometry model and −14.15 kJ mol^–1^ for the fixed 1:1 model). In addition, the calculated thermodynamic parameters indicate that the favorable Δ*G*° associated with the inclusion process is predominantly driven by entropy.

Regarding the enthalpic contribution, a small negative Δ*H*° value was obtained (−1.71 and −3.47 kJ mol^–1^, for the variable and fixed stoichiometry models, respectively). This exothermic contribution can be attributed to the formation of a favorable intermolecular interaction between the host and guest species during the inclusion process. The enthalpy change reflects the balance between favorable and unfavorable energetic contributions, including van der Waals interactions, hydrogen bonding, and the hydrophobic interactions, together with the energetic cost associated with the partial dehydration of the βCD cavity and the guest molecule upon complex formation. The observed entropic gain could be attributed not only to the formation of higher-order supramolecular assemblies in the variable stoichiometry model but also to the release of water molecules originally located within the βCD cavity and the concomitant dehydration of both the host and guest molecules.^10^

### 3.2 Isothermal Titration Calorimetry: CM-βCD

In order to investigate whether the interaction with SEL could be improved using a modified CD, the supramolecular interaction between SEL and CM-βCD was also investigated by ITC. CM-βCD has been shown to be a host molecule capable of enhancing host–guest interactions^36^ due to its similar cavity size compared with native βCD, combined with its ability to establish ionic interactions through its carboxymethyl substituents (particularly for the sodium salt form of CM-βCD).^37^ Therefore, ITC titrations between CM-βCD and SEL were carried out at four different temperatures (from 298.15 to 328.15 K) to evaluate the temperature dependence of the complexation process. The thermodynamic parameters obtained from these titrations are presented in Table 3.

**Table 3:** Thermodynamic Parameters Describing the Supramolecular Interaction between CM-βCD and SEL in Aqueous Solution at Different Temperatures

*T*/K	*n*	*K*_eq_	Δ*H*° / kJ mol^–1^	*T*Δ*S*° / kJ mol^–1^	Δ*G*° / kJ mol^–1^
298.15	1.42 ± 0.03	3,500 ± 224	–2.43 ± 0.43	17.84 ± 0.35	–20.23 ± 0.01
308.15	1.39 ± 0.09	3,665 ± 92	–3.32 ± 0.44	17.66 ± 0.36	–20.98 ± 0.07
318.15	1.46 ± 0.02	3,135 ± 64	–4.71 ± 0.54	16.06 ± 1.19	–20.77 ± 0.65
328.15	1.54 ± 0.11	3,240 ±255	–4.34 ± 1.16	17.71 ± 0.97	–22.05 ± 0.28
298.15	1.00	1,050 ± 71	–5.48 ± 0.43	11.76 ± 0.27	–17.24 ± 0.16
308.15	1.00	1,305 ± 78	–6.48 ± 0.17	11.89 ± 0.01	–18.37 ± 0.16
318.15	1.00	1,026 ± 133	–10.37 ± 0.42	7.70 ± 0.07	–18.07 ± 0.35
328.15	1.00	963 ± 293	–10.64 ± 0.85	8.03 ± 0.01	–18.67 ± 0.84

Similarly to the βCD:SEL supramolecular system, the stoichiometry was determined, and a value of *n* ≈ 1.5 was obtained for all four temperatures investigated. The consistent observation of this non-integer stoichiometry across the investigated temperature range suggests that the interaction between SEL and both βCD and CM-βCD involves the formation of higher-order supramolecular assemblies rather than simple 1:1 host–guest complexes, as previously reported for other guest molecules.^15^ Accordingly, the thermodynamic parameters for CM-βCD:SEL supramolecular system were also determined using two fitting approaches: (i) allowing all fitting parameters to vary freely and (ii) fixing the host-to-guest stoichiometry at 1:1, following the same procedure adopted for the βCD:SEL system. The resulting ITC titration curves for both fitting models are shown in Figures 3 and 4, respectively.

**Figure 3:**
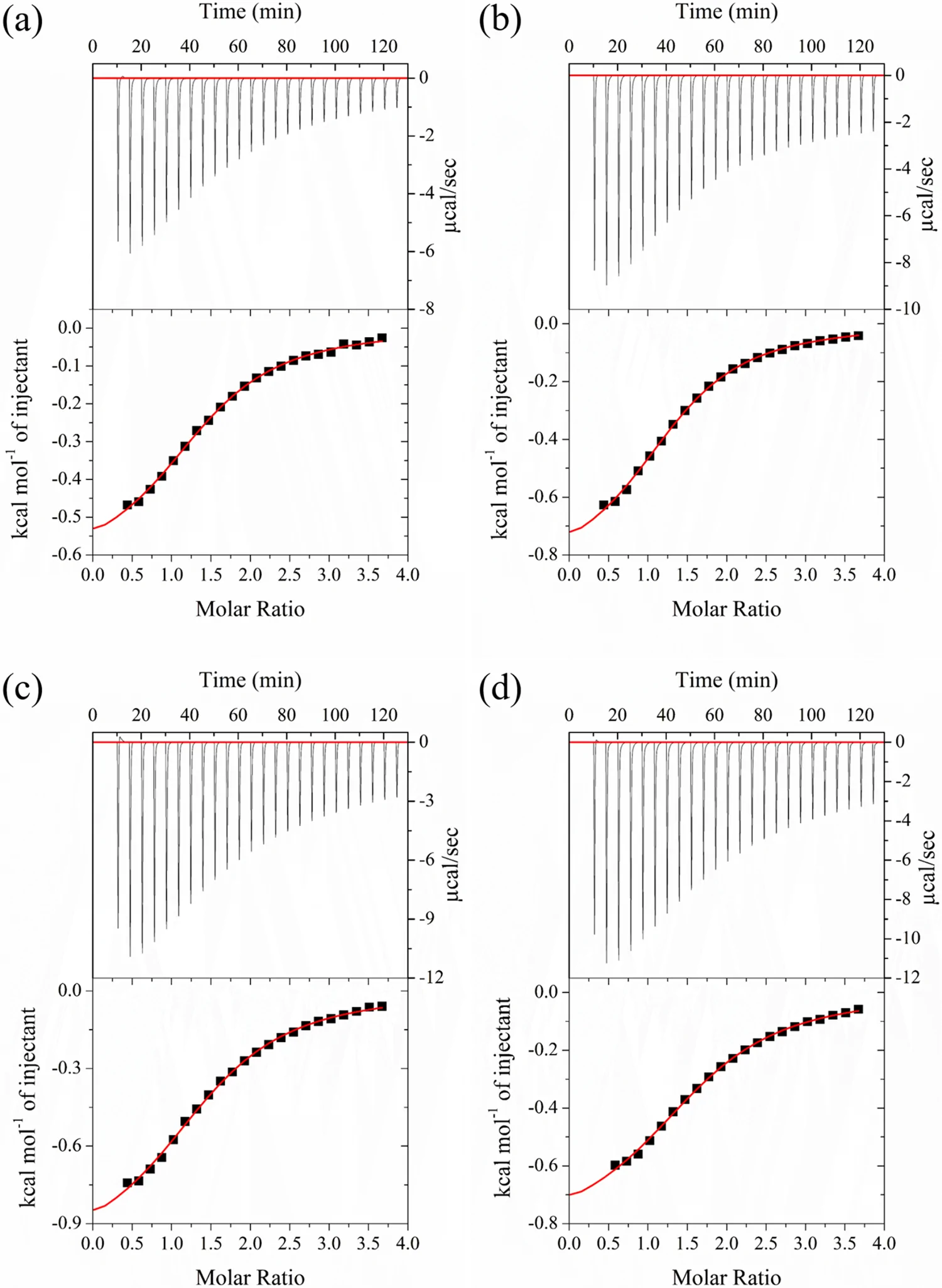
ITC titration curves for CM-βCD:SEL supramolecular system at: (a) 298.15 K, (b) 308.15 K, (c) 318.15 K, and (d) 328.15 K. Stoichiometry was not fixed for all nonlinear fitting.

**Figure 4:**
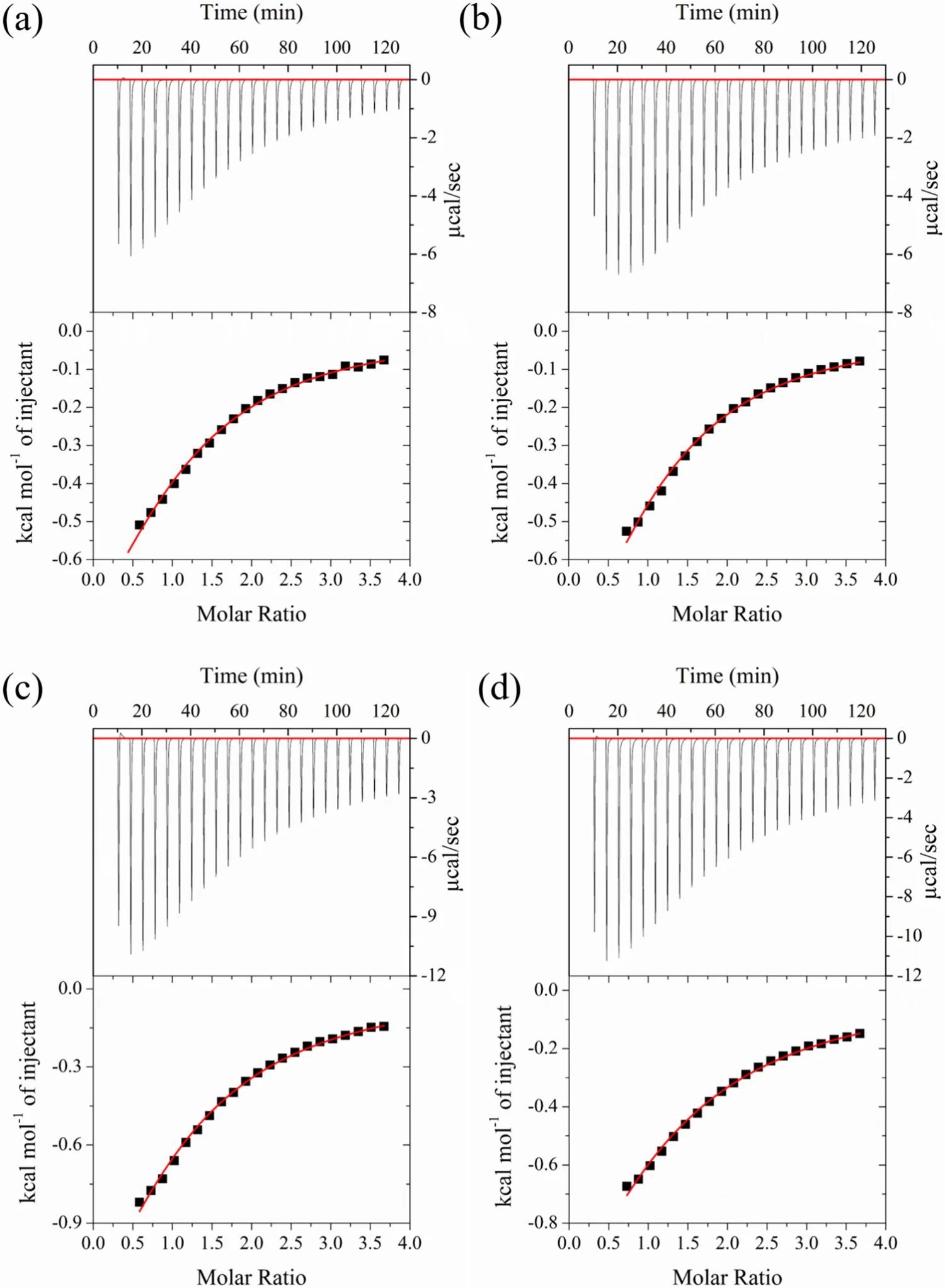
ITC titration curves for CM-βCD:SEL supramolecular system at: (a) 298.15 K, (b) 308.15 K, (c) 318.15 K, and (d) 328.15 K. Stoichiometry was fixed at 1:1 molar ratio for all nonlinear fitting.

The supramolecular interaction between CM-βCD and SEL was spontaneous at all investigated temperatures, with the *K*_eq_ values consistently higher than those observed for the βCD:SEL system. Similarly to the βCD system, fixing the stoichiometry at 1:1 resulted in lower *K*_eq_ values (1305–963) than those obtained when all fitting parameters were allowed to vary (*K*_eq_ 3665–3135). However, the *K*_eq_ obtained using the fixed stoichiometry model are comparable to those reported for several CD-based supramolecular systems, for which *K*_eq_ values are typically on the order of 1.000.^38,39^ However, these values remain lower than those reported for adamantane derivatives, which are well-established high-affinity guests for βCD and its derivatives.^40^

The stronger binding affinity of the CM-βCD:SEL system is directly reflected in the more favorable Δ*G*° value observed at 298.15 K (−20.23 kJ mol^–1^), compared with that of the native β-CD:SEL system (−15.55 kJ mol^–1^). For the variable stoichiometry model, the inclusion process was entropy-driven (|Δ*S*°| > |Δ*H*°|). However, when the stoichiometry was fixed (*n* = 1), the inclusion process remained entropy-driven at 298.15 and 308.15 K, whereas enthalpic contributions became dominant at 318.15 and 328.15 K (Table 3). For both fitting models, the heat capacity change (ΔCp) was calculated (Figure S3). The calculated ΔCp values were −0.094 kJ mol^–1^.K^–1^ for the variable stoichiometry model and −0.229 kJ mol^–1^.K^–1^ for the fixed stoichiometry model. These negative ΔCp values are consistent with the transfer of the hydrophobic regions of the guest molecule from the aqueous medium to the hydrophobic environment of the host, accompanied by solvent reorganization during complex formation. Accordingly, the larger negative ΔCp observed for the fixed stoichiometry model is consistent with the greater enthalpic contribution identified for this fitting approach (Table 3).

### 3.3 Nuclear Magnetic Resonance

NMR techniques were employed in this study to characterize and assign the structures of the SEL guest molecule and its IC with the CM-βCD host, which exhibited the highest *K*_eq_ values among the investigated host–guest systems. ^1^H NMR experiments were carried out using the NOESYGPPR1D pulse sequence, and the ^1^H and ^13^C NMR chemical shift assignments of SEL were performed by combining 1D and 2D experiments at different temperatures, owing to the molecular structure of SEL, which affected the spectral resolution. The best results for SEL were achieved at 50 °C, which enabled the resolution of overlapping resonances observed in the spectrum acquired at 25 °C. The ^1^H and ^13^C chemical shifts for the free guest molecule are presented in Table S2.

In order to evaluate the host–guest interaction between CM-βCD and SEL, 2D ^1^H/^1^H ROESY experiments were performed. Figure 5a,b shows the ^1^H NMR spectrum and 2D ^1^H/^1^H ROESY spectrum of the CM-βCD:SEL supramolecular system in D_2_O, respectively. Although comparisons of ^1^H NMR chemical shifts between the free guest and the IC are commonly used to evaluate host–guest interactions, such an analysis was not carried out in the present study because different solvents and acquisition temperatures were employed for the free SEL and CM-βCD:SEL samples, which would preclude a reliable comparison of the chemical shift variations.

**Figure 5:**
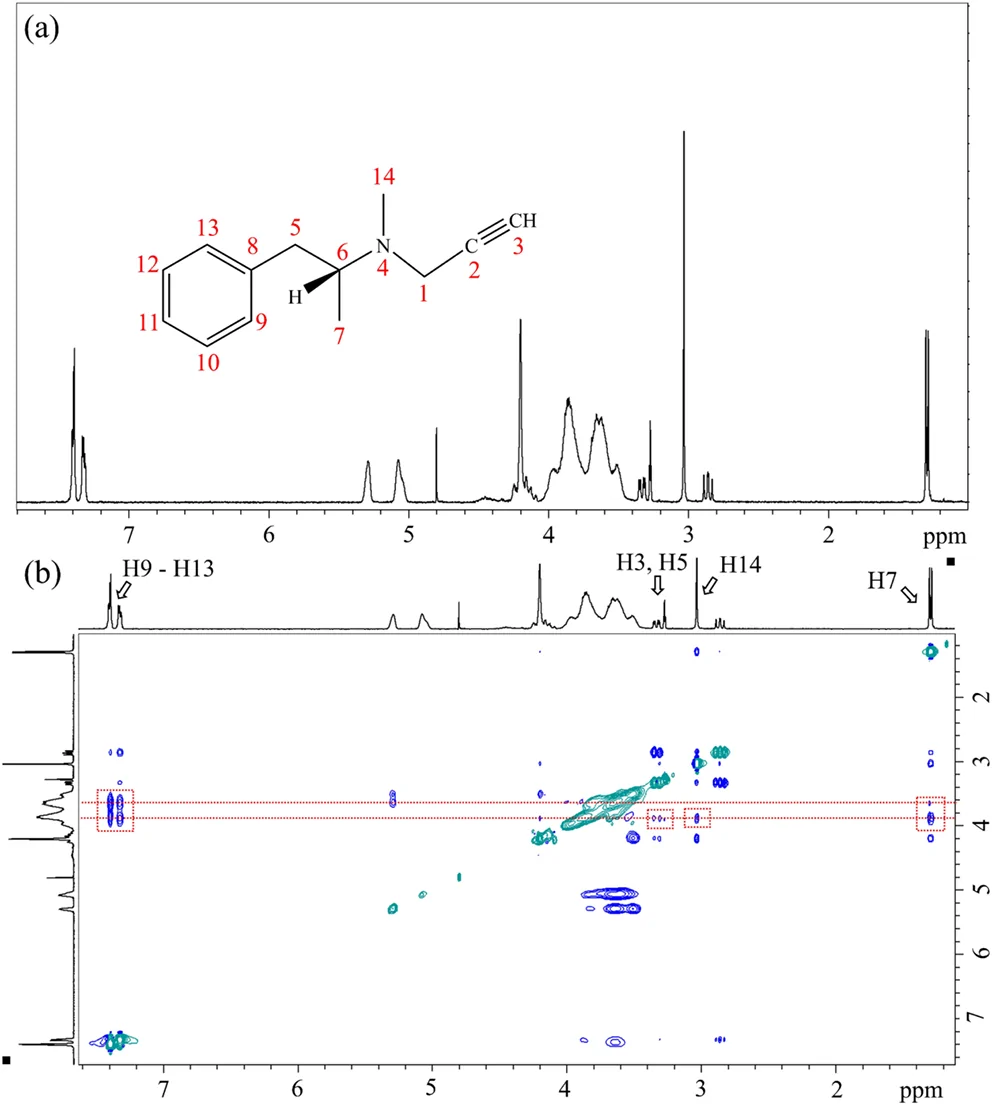
(a) ^1^H NMR spectrum (400 MHz) of CM-βCD:SEL supramolecular complex in D_2_O and (b) 2D ^1^H/^1^H ROESY spectrum (400 MHz) of CM-βCD:SEL supramolecular complex in D_2_O.

However, clear cross-peak correlations between the SEL and CM-βCD hydrogens can be observed in the 2D ROESY contour map (Figure 5b). These data demonstrate strong spatial correlations between the aromatic protons of SEL (H9–H13) and both internal and external hydrogens from the CM-βCD. Similarly, hydrogen H7 of SEL presents strong cross-peak correlations with the internal CM-βCD hydrogens. In addition, weaker cross-peak correlations involving the internal CM-βCD hydrogens were verified for the SEL hydrogens H3, H5, and H14. Overall, these data suggest a deep inclusion of SEL within the CM-βCD cavity. Moreover, the simultaneous observation of multiple host–guest contacts supports the existence of higher-order supramolecular assemblies, consistent with the non-integer stoichiometry (*n* ≈ 1.5) obtained from the ITC experiments, in which three host molecules interact with two guest molecules. To further elucidate the supramolecular organization of these ICs, MD simulations were performed, considering both the 1:1 inclusion model (fixed stoichiometry) and the proposed higher-order assembly (3:2 host-to-guest stoichiometry).

### 3.4 Theoretical Calculations

At first moment, the IC with a 1:1 molar ratio between CM-βCD and SEL was investigated. During the simulation, the distance between the two molecules fluctuated (Movie S1). As the simulation progressed, spontaneous complex formation was observed, followed by the insertion of SEL into the CM-βCD cavity. Figure 6a shows a representative snapshot from the MD simulation, presenting the structure of CM-βCD:SEL supramolecular complex, in which the aromatic ring of the guest is preferentially included within the host cavity. The preferential inclusion of the aromatic ring of SEL into the CM-βCD cavity through its wider rim is consistent with previous studies on CDs-based supramolecular systems, in which this moiety is preferentially included in the CDs cavities.^41,42^

**Figure 6:**
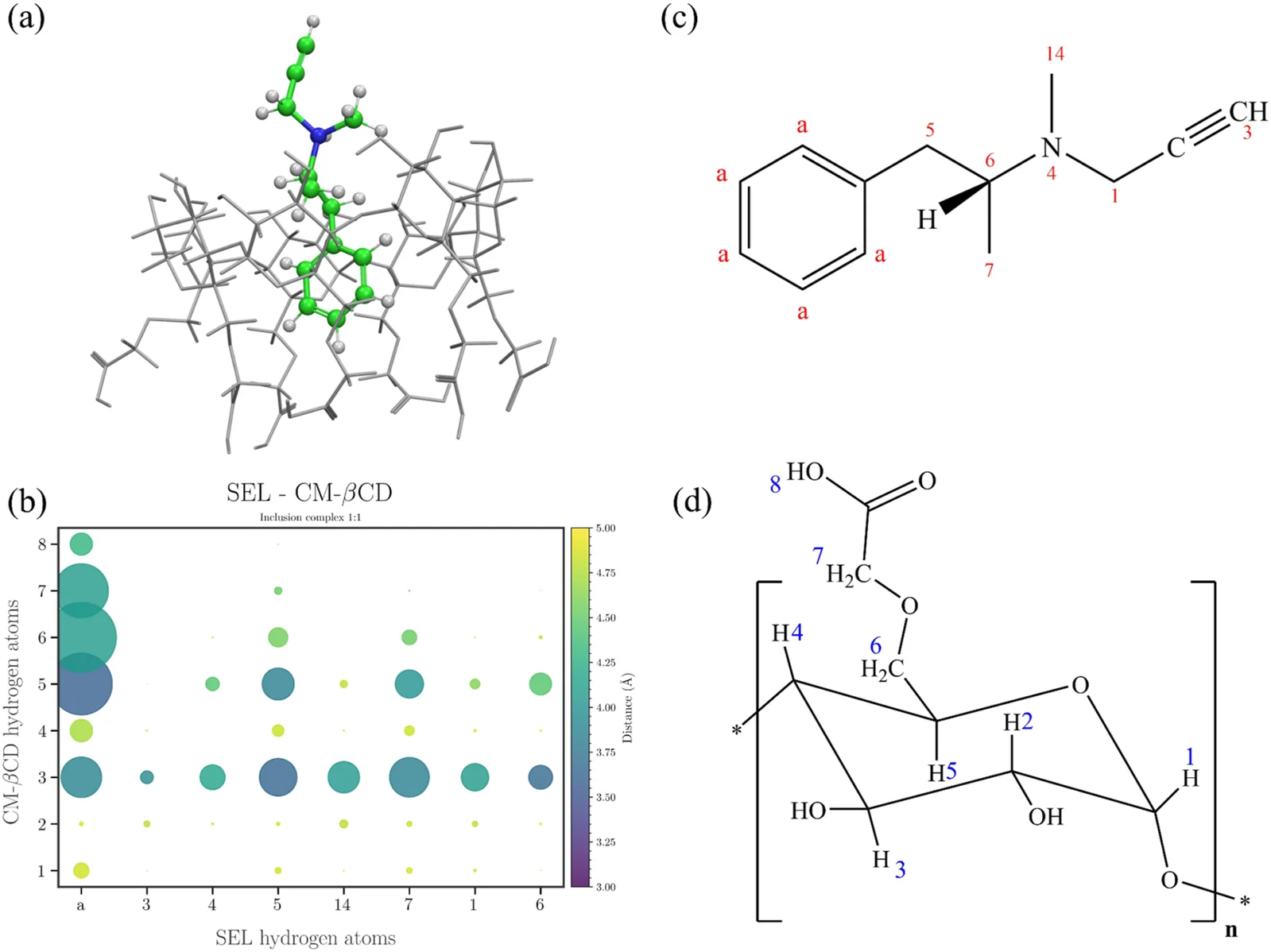
(a) CM-βCD:SEL supramolecular complex obtained by MD simulation at 1:1 molar ratio, (b) calculated correlation distances between SEL and CM-βCD hydrogens, (c) numbered SEL guest molecule, and (d) numbered CM-βCD host molecule.

In addition to the MD simulation for the CM-βCD:SEL IC, hydrogen distances between the host and guest molecules were calculated.^33^ These data are comparable with 2D ^1^H/^1^H NMR ROESY experiments, allowing the identification of short-range spatial correlations between host and guest hydrogens, as well as the frequency with which these short-range interactions occurred during the MD simulation (Figure 6b). The calculated distance maps reveal that the aromatic hydrogens of SEL (H9–H13) exhibit frequent short-range contacts with both the internal and external hydrogens of CM-βCD, in agreement with the experimental 2D ^1^H/^1^H NMR ROESY data. In addition, SEL hydrogens H5 and H7 showed significant correlations with internal CM-βCD hydrogens (H3 and H5). Likewise, SEL hydrogens H1, H4, and H14 displayed weaker interactions with the internal CM-βCD hydrogen H3. These data are consistent with those obtained experimentally from the 2D NMR approach.

Analysis of the MD trajectories further revealed that SEL undergoes repeated association and dissociation events with the CM-βCD cavity throughout the simulation, as illustrated in Figure S4. This dynamic behavior is also reflected in the non-radial distribution of short-range contacts shown in Figure S5. Despite these fluctuations, the guest molecule remained inside the CM-βCD cavity for approximately 80% of the total simulation time, indicating a stable inclusion process under dynamic equilibrium conditions. Another result supporting this behavior is the strong interaction between CM-βCD and the surrounding water molecules, reflected by an average interaction potential energy of −183,277 kJ mol^–1^. This observation highlights the complexity of the system and suggests that host–guest complexation occurs within a dynamic multiple-equilibrium environment strongly influenced by solvent interactions. To evaluate the structural feasibility of the higher-order supramolecular assembly suggested by the ITC experiments (*n* ≈ 1.5), a more complex molecular model was subsequently constructed, as shown in Figure 7a.

**Figure 7:**
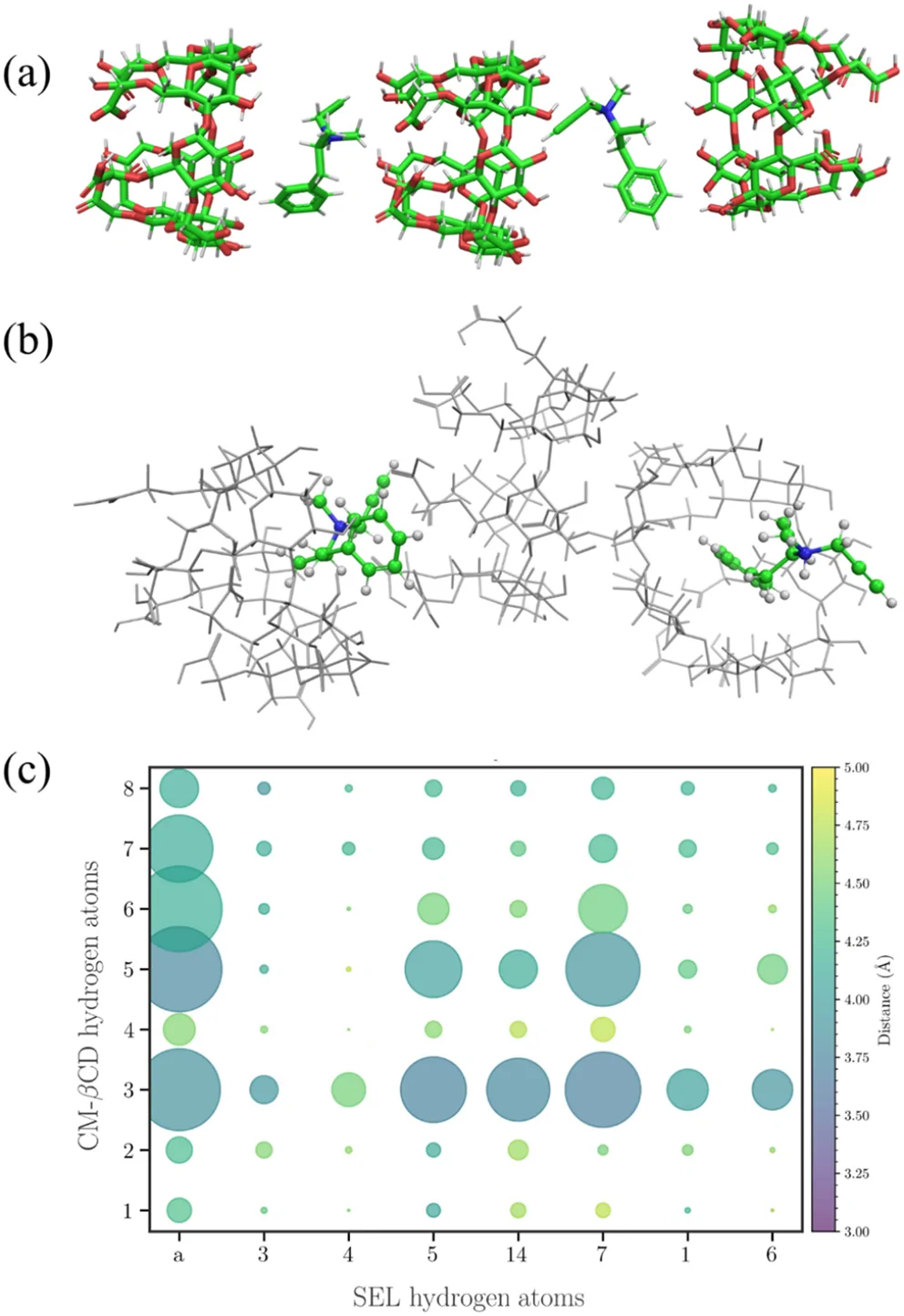
(a) structural model of a complex considering a ratio 3:2, (b) complex 3:2 CM-βCD:SEL formed, and (c) correlation distances between SEL and CM-βCD hydrogens in complex 3:2 (membered SEL and CM-βCD structures as presented in Figure 6).

To evaluate the feasibility of the proposed 3:2 host-to-guest supramolecular assembly, an additional MD simulation was performed on the basis of the stoichiometry suggested by the experimental results. After completing the MD simulation, the trajectory was extracted, revealing the presence of the complex, as shown in Figure 7b. In addition, hydrogen distances were calculated to verify whether the same spatial correlations observed for the 1:1 (Figure 6b) were also preserved in the higher-order assembly. Figure 7c demonstrates that the characteristic host–guest correlations identified for the 1:1 complex are likewise present in the 3:2 assembly. Moreover, these correlations occur with higher frequency and greater intensity, indicating a more stable and persistent network of intermolecular contacts than that observed for the conventional 1:1 IC, demonstrating that an equilibrium between the conventional 1:1 and 3:2 supramolecular systems can occur (Figure 8).

**Figure 8:**
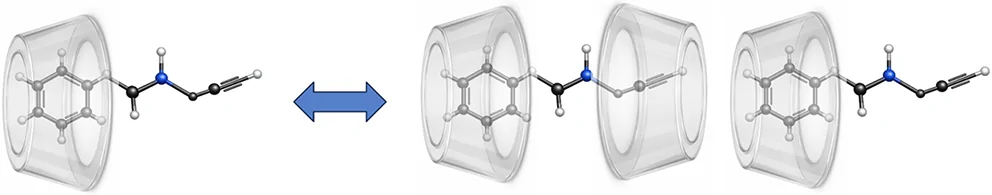
Schematic representation of both supramolecular systems formed between CDs (βCD or CM-βCD) with SEL guest molecule.

## 4 Conclusion

The supramolecular interactions between SEL and CDs derivatives were comprehensively investigated through a combined thermodynamic, spectroscopic, and theoretical approach. ITC demonstrated that both βCD and CM-βCD form spontaneous ICs with SEL in aqueous solution over the investigated temperature range, with CM-βCD presenting significantly higher equilibrium constants and more favorable Gibbs free energies of interaction. The thermodynamic profiles revealed a strong contribution from entropic effects, indicating that solvent reorganization, dehydration processes, and hydrophobic interactions play central roles in the complexation mechanism.

Importantly, the calorimetric analyses consistently produced non-integer stoichiometric values (*n* ≈ 1.5), suggesting that the supramolecular organization of these systems cannot be adequately described by a simple 1:1 host–guest equilibrium model. The negative heat capacity values further support extensive solvent restructuring and hydrophobic transfer upon complex formation, reinforcing the dynamic multi-equilibrium behavior of these assemblies in aqueous solution.

The 2D ^1^H/^1^H NMR ROESY experiments demonstrated pronounced spatial correlations between the aromatic region of SEL and both internal and external CM-βCD hydrogens, indicating deep cavity inclusion and the presence of extended intermolecular interactions. These experimental observations were corroborated by MD simulations, which revealed stable inclusion events, dynamic host–guest exchange behavior, and the feasibility of higher-order supramolecular arrangements. In particular, the theoretical analyses support the formation of a 3:2 CM-βCD:SEL assembly, whose interaction patterns and structural organization are consistent with the experimental spectroscopic and thermodynamic data.

Overall, the combined results provide strong thermodynamic and structural evidence for the existence of higher-order CD supramolecular assemblies in aqueous solution. The excellent agreement between ITC, ROESY, and MD simulations demonstrates that the observed non-integer stoichiometry arises from a dynamic multi-equilibrium system rather than a simple 1:1 inclusion process. In addition, these findings contribute to a broader molecular-level understanding of how modified CDs can mediate cooperative host–guest interactions, solvent organization, and supramolecular topology in aqueous media.

## Supplementary Material





## Notes

1H NMR spectrum of CM-βCD, ^1^H and ^13^C chemical shifts for SEL, optimized structures for SEL and CM-βCD, heat capacities for CM-βCD:SEL, calculated distances between the SEL center of mass and CM-βCD:SEL over the 1:1 MD simulation, and radial distribution function of the 1:1 CM-βCD:SEL (PDF)
During the simulation, the distance between the two molecules fluctuated (MP4)
