# Supplementary material for: Thermodynamic and Structural Evidence for Higher-Order Selegiline with Carboxymethyl-β-Cyclodextrin Supramolecular Assemblies in Aqueous Solution
Source: J Phys Chem B. 2026 Sep 6;130(37):9594–604. doi: 10.1021/acs.jpcb.6c03442 (PMC13592018; doi:10.1021/acs.jpcb.6c03442)
Supplement: Supplementary file 1 [file jp6c03442_si_001.pdf]

**Supporting Information**

# Thermodynamic and Structural Evidence for Higher-Order Selegiline with Carboxymethyl-β-Cyclodextrin Supramolecular Assemblies in Aqueous Solution

*Diego Caproni de Morais[a], Rogério R. Macedo[b], Bianca B. M. Vieira[a], Ivana Lula[c], Juliana Fedoce Lopes[b*], and Frederico B. De Sousa[a*]*

[a]Laboratório de Sistemas Poliméricos e Supramoleculares (LSPS) - Instituto de Física e Química, Universidade Federal de Itajubá (UNIFEI), Itajubá, 37500-903, MG, Brazil.

[b]Laboratório de Química Computacional (LaQC) – Instituto de Física e Química, Universidade Federal de Itajubá (UNIFEI), Itajubá, 37500-903, MG, Brazil.

[c]Departamento de Química – Instituto de Ciências Exatas, Universidade Federal de Minas Gerais (UFMG), Belo Horizonte 31270-901, MG, Brazil.

Corresponding author:
Prof. Frederico B. De Sousa
Laboratório de Sistemas Poliméricos e Supramoleculares (LSPS) - Instituto de Física e Química, Universidade Federal de Itajubá (UNIFEI), Itajubá, 37500-903, MG, Brazil.
55 35 3629175
fredbsousa@gmail.com, fredbsousa@unifei.edu.br

Prof. Juliana Fedoce Lopes
Laboratório de Química Computacional (LaQC) – Instituto de Física e Química, Universidade Federal de Itajubá (UNIFEI), Itajubá, 37500-903, MG, Brazil
55 35 3629175
jfedoce@unifei.edu.br

Supporting Information (Figure S1)

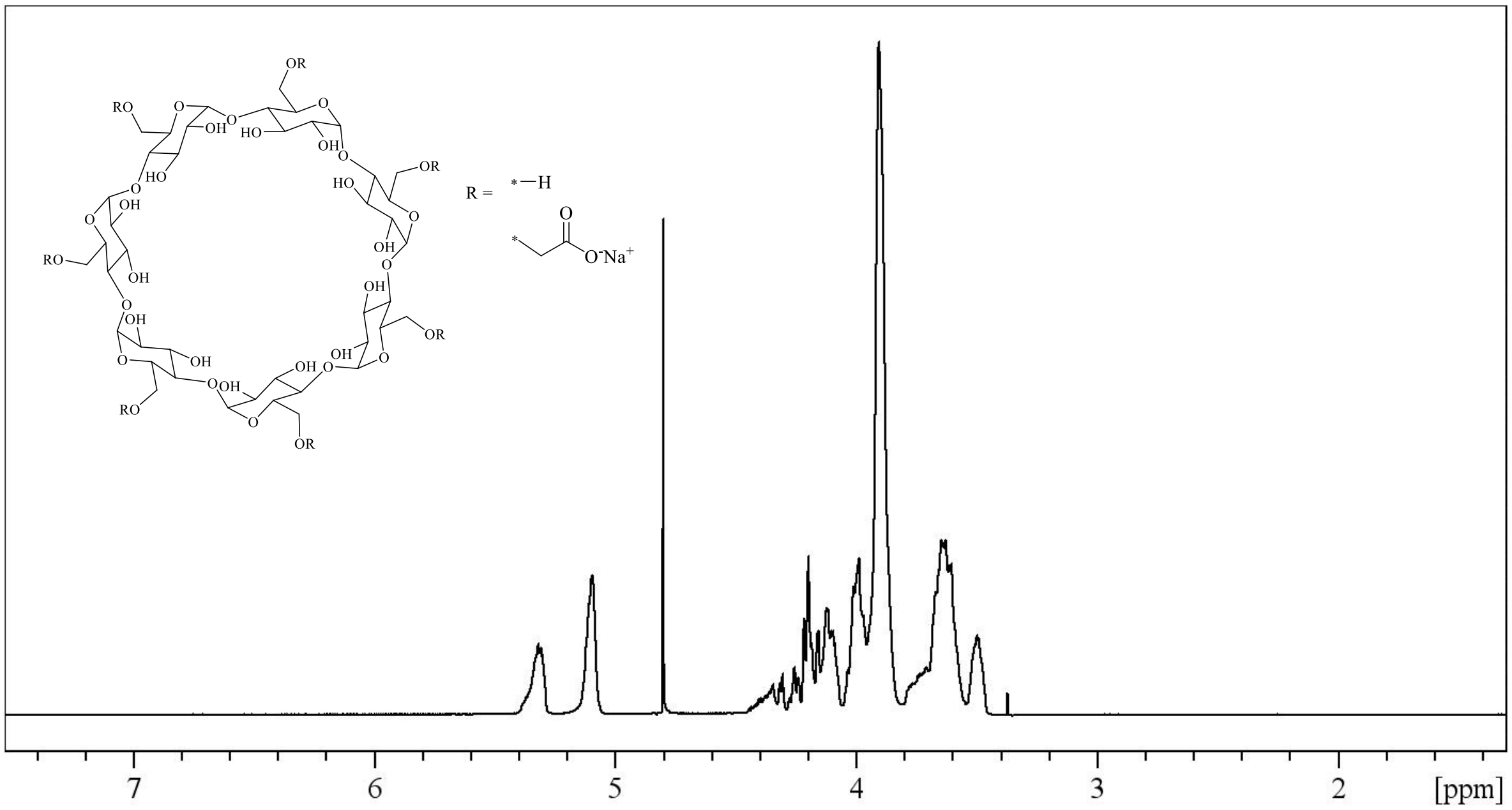


Figure S1: ¹H NMR spectrum of CM-βCD in $D_2O$ at 400 MHz.

Supporting Information (Figure S2)

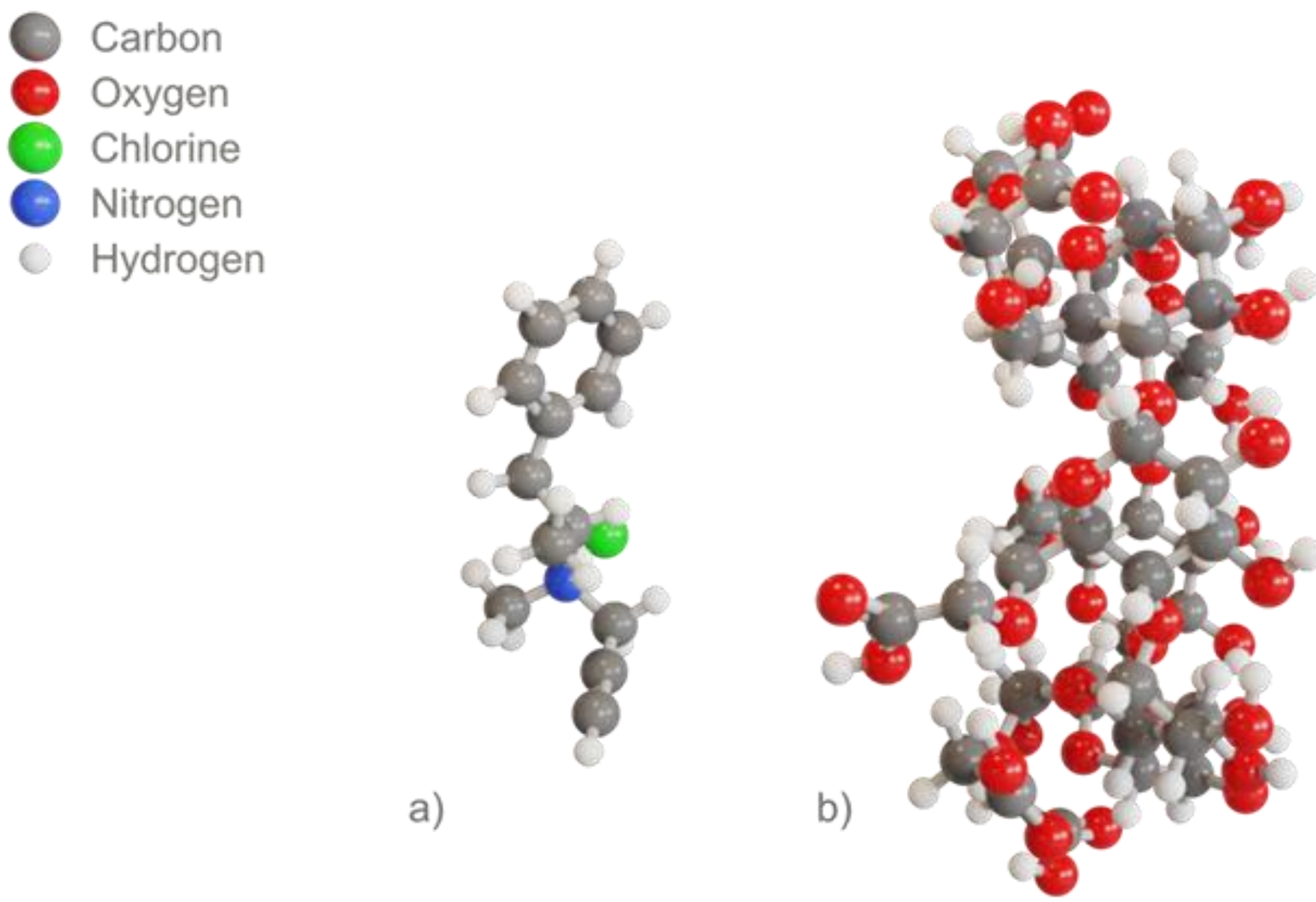


a) b)

Figure S2: Optimized structures of: a) selegiline hydrochloride and b) carboxymethyl-β-cyclodextrin.

Supporting Information (Table S1)

Table S1a: Cartesian coordinates of the optimized structure for SEL: OPT_B3LYP_631+G(d,p).

E = -1021,1070 Hartree, $\nu_{(min)}$ = 26.68 $cm^{-1}$

| | | | |
|---|---|---|---|
| Cl | -1.27200 | 2.98000 | 0.01800 |
| N | -1.80500 | 0.15900 | 0.26600 |
| C | -4.32100 | -2.13500 | -0.89700 |
| C | -3.61700 | -1.15400 | -0.86500 |
| C | -2.81300 | 0.06300 | -0.84600 |
| C | -0.47700 | -0.52100 | -0.01800 |
| C | 0.58300 | -0.02700 | 0.99700 |
| C | 1.98700 | -0.12300 | 0.43200 |
| C | 2.84500 | -1.17700 | 0.76700 |
| C | 4.12800 | -1.25500 | 0.22100 |
| C | 4.56900 | -0.27400 | -0.66800 |
| C | 3.72200 | 0.78600 | -1.00400 |
| C | 2.44200 | 0.86300 | -0.45800 |
| C | -2.40500 | -0.10600 | 1.59800 |
| C | -0.56700 | -2.04300 | -0.09500 |
| H | -1.56100 | 1.26900 | 0.23600 |
| H | -4.93800 | -3.00400 | -0.93200 |
| H | -2.26200 | 0.19300 | -1.78300 |
| H | -3.45000 | 0.94900 | -0.73700 |
| H | -0.20100 | -0.12000 | -0.99800 |
| H | 0.36800 | 1.02000 | 1.23800 |
| H | 0.51300 | -0.61200 | 1.92200 |
| H | 2.51100 | -1.93800 | 1.47000 |
| H | 4.78300 | -2.07800 | 0.49500 |
| H | 5.56800 | -0.33100 | -1.09200 |
| H | 4.06300 | 1.56000 | -1.68600 |

| | | | |
|---|---|---|---|
| H | 1.78800 | 1.69700 | -0.70500 |
| H | -2.76500 | -1.13400 | 1.66500 |
| H | -1.65900 | 0.09200 | 2.36700 |
| H | -3.23800 | 0.58800 | 1.73300 |
| H | 0.42600 | -2.43500 | -0.33600 |
| H | -0.87400 | -2.48000 | 0.86200 |
| H | -1.26400 | -2.38300 | -0.86600 |

Table S1b: Cartesian coordinates of the optimized structure for CM-βCD: OPT_M06-2X_631G(d,p).

E = 5,868.3531 Hartree, $\nu_{(min)}$ = 36.58 $cm^{-1}$

| | | | |
|---|---|---|---|
| C | 0.72500 | 5.91600 | -0.48200 |
| C | 1.62800 | 6.11500 | -1.70200 |
| C | 2.31700 | 4.79900 | -2.05400 |
| C | 3.04600 | 4.27700 | -0.83600 |
| C | 2.05600 | 4.11700 | 0.31900 |
| C | 2.71400 | 3.59900 | 1.58600 |
| C | -4.37500 | 4.55700 | -0.49300 |
| C | -3.89100 | 5.35700 | -1.70400 |
| C | -2.41000 | 5.07100 | -1.92900 |
| C | -1.63400 | 5.38900 | -0.66800 |
| C | -2.21500 | 4.60000 | 0.50800 |
| C | -1.50400 | 4.92100 | 1.81600 |
| C | -6.22000 | -0.40900 | -0.63000 |
| C | -6.56900 | 0.49500 | -1.81100 |
| C | -5.47500 | 1.54600 | -1.95800 |
| C | -5.34800 | 2.33400 | -0.67300 |
| C | -5.09500 | 1.38200 | 0.50100 |
| C | -5.13300 | 2.09900 | 1.84000 |
| C | -3.29100 | -4.83300 | -0.63300 |
| C | -4.33200 | -4.62100 | -1.73100 |
| C | -4.52000 | -3.13200 | -1.98100 |
| C | -4.90800 | -2.45000 | -0.68600 |
| C | -3.80700 | -2.68200 | 0.34800 |
| C | -4.17200 | -2.14900 | 1.72500 |
| C | 1.81200 | -5.95900 | -0.75000 |
| C | 1.02900 | -6.39800 | -1.98800 |
| C | -0.14100 | -5.44000 | -2.20500 |
| C | -0.98000 | -5.39500 | -0.94700 |
| C | -0.11700 | -5.00000 | 0.25500 |

| | | | |
|---|---|---|---|
| C | -0.90000 | -5.00300 | 1.55700 |
| C | 5.73600 | -2.39000 | -0.77200 |
| C | 5.65300 | -3.33300 | -1.97100 |
| C | 4.20400 | -3.77800 | -2.13500 |
| C | 3.71500 | -4.44000 | -0.86500 |
| C | 3.91900 | -3.50100 | 0.32800 |
| C | 3.61900 | -4.18600 | 1.65000 |
| C | 4.98600 | 2.86600 | -0.73700 |
| C | 5.76500 | 2.25000 | -1.89600 |
| C | 5.29300 | 0.82000 | -2.11700 |
| C | 5.47100 | 0.02500 | -0.83900 |
| C | 4.67200 | 0.69500 | 0.27900 |
| C | 4.90900 | 0.07400 | 1.64800 |
| O | 0.93700 | 6.62400 | -2.81000 |
| O | 3.26100 | 4.98100 | -3.08600 |
| O | 3.66000 | 3.05000 | -1.17300 |
| O | 1.45700 | 5.38100 | 0.60400 |
| O | 3.95100 | 4.21600 | 1.88700 |
| O | -4.63100 | 5.09400 | -2.86400 |
| O | -1.90100 | 5.86900 | -2.97600 |
| O | -0.28600 | 5.01800 | -0.87900 |
| O | -3.61000 | 4.88400 | 0.65100 |
| O | -1.17500 | 6.28700 | 1.96900 |
| O | -6.72600 | -0.22000 | -3.00900 |
| O | -5.78100 | 2.44500 | -3.00400 |
| O | -4.23100 | 3.20000 | -0.81300 |
| O | -6.07400 | 0.34400 | 0.55700 |
| O | -6.34700 | 2.78100 | 2.08900 |
| O | -3.97600 | -5.28600 | -2.91600 |
| O | -5.53700 | -2.90100 | -2.93100 |
| O | -5.02100 | -1.06100 | -0.94900 |
| O | -3.57200 | -4.07500 | 0.51900 |
| O | -5.31000 | -2.77100 | 2.28300 |
| O | 1.82300 | -6.48400 | -3.13900 |
| O | -0.96600 | -5.88300 | -3.26100 |
| O | -2.03800 | -4.47700 | -1.15800 |
| O | 0.96400 | -5.92300 | 0.38400 |
| O | -1.73600 | -6.14900 | 1.66600 |
| O | 6.11600 | -2.73800 | -3.15600 |
| O | 4.07900 | -4.70000 | -3.19700 |
| O | 2.32400 | -4.68300 | -1.02200 |
| O | 5.26300 | -3.02600 | 0.40000 |
| O | 4.38300 | -5.35400 | 1.87500 |

| | | | |
|---|---|---|---|
| O | 5.63400 | 3.00600 | -3.07500 |
| O | 6.03600 | 0.19500 | -3.14100 |
| O | 4.94800 | -1.27300 | -1.07400 |
| O | 5.02400 | 2.06500 | 0.41300 |
| O | 6.24700 | 0.14600 | 2.08800 |
| H | 0.29500 | 6.85600 | -0.12400 |
| H | 2.40500 | 6.84700 | -1.44300 |
| H | 1.55000 | 4.06700 | -2.35300 |
| H | 3.81200 | 5.01100 | -0.54600 |
| H | 1.27700 | 3.39800 | 0.02500 |
| H | 2.01300 | 3.71800 | 2.42600 |
| H | 2.94700 | 2.54200 | 1.45400 |
| H | -5.41400 | 4.79200 | -0.24000 |
| H | -4.00500 | 6.42600 | -1.48300 |
| H | -2.28600 | 4.00200 | -2.16200 |
| H | -1.70300 | 6.46700 | -0.46700 |
| H | -2.08900 | 3.52500 | 0.30600 |
| C | -2.25000 | 7.14600 | 2.22000 |
| H | -7.02100 | -1.12900 | -0.43200 |
| H | -7.51700 | 1.00100 | -1.59200 |
| H | -4.51800 | 1.04000 | -2.15800 |
| H | -4.09500 | 0.94000 | 0.36800 |
| H | -4.35500 | 2.86800 | 1.84800 |
| H | -4.93400 | 1.37200 | 2.64200 |
| H | -3.29300 | -5.87300 | -0.29600 |
| H | -5.28400 | -5.04000 | -1.38100 |
| H | -3.56500 | -2.70400 | -2.32900 |
| H | -5.85200 | -2.86300 | -0.30500 |
| H | -2.88900 | -2.18000 | 0.00100 |
| H | -3.30700 | -2.26600 | 2.39700 |
| H | -4.41700 | -1.08700 | 1.63600 |
| H | 2.61500 | -6.66200 | -0.50500 |
| H | 0.62000 | -7.40000 | -1.80300 |
| H | 0.25600 | -4.43500 | -2.41400 |
| H | -1.38700 | -6.39900 | -0.77000 |
| H | 0.28100 | -3.98600 | 0.09500 |
| H | -0.20200 | -4.94800 | 2.40300 |
| H | -1.56000 | -4.13500 | 1.57700 |
| H | 6.77000 | -2.10100 | -0.55900 |
| H | 6.27500 | -4.21400 | -1.77000 |
| H | 3.58400 | -2.88900 | -2.32700 |
| H | 4.26100 | -5.37700 | -0.70200 |
| H | 3.23200 | -2.64800 | 0.20900 |

| | | | |
|---|---|---|---|
| H | 3.77100 | -3.46700 | 2.46900 |
| H | 2.57500 | -4.51200 | 1.64500 |
| H | 5.43000 | 3.82000 | -0.43500 |
| H | 6.82600 | 2.23000 | -1.62000 |
| H | 4.22100 | 0.83300 | -2.37400 |
| H | 6.53300 | -0.01400 | -0.56300 |
| H | 3.60100 | 0.60000 | 0.03100 |
| H | 4.66400 | -0.99100 | 1.59800 |
| H | 4.24400 | 0.56500 | 2.37600 |
| H | -0.54100 | 4.40600 | 1.82000 |
| H | -6.26800 | 2.90400 | -0.49900 |
| H | 0.07000 | 6.18500 | -2.88200 |
| H | 2.82800 | 5.52400 | -3.76000 |
| C | 3.85000 | 5.51200 | 2.39300 |
| H | -4.82900 | 4.14000 | -2.92300 |
| H | -2.55200 | 5.83300 | -3.69100 |
| H | -6.12300 | -0.98600 | -3.01400 |
| H | -6.11400 | 1.90900 | -3.73800 |
| C | -7.44000 | 1.95700 | 2.39300 |
| H | -3.01300 | -5.21300 | -3.04200 |
| H | -5.39600 | -3.54000 | -3.64500 |
| C | -5.08800 | -4.06200 | 2.79600 |
| H | 2.45400 | -5.73900 | -3.16500 |
| H | -0.37100 | -6.14400 | -3.97900 |
| H | 5.86800 | -1.79500 | -3.16600 |
| H | 4.62100 | -4.35600 | -3.92200 |
| C | 5.73100 | -5.13200 | 2.18900 |
| H | 4.73800 | 3.38400 | -3.11000 |
| H | 6.12400 | 0.84600 | -3.85200 |
| C | 6.68500 | 1.42800 | 2.47700 |
| H | -2.11300 | 4.55800 | 2.65700 |
| H | -5.76500 | -4.19300 | 3.64900 |
| H | -4.05500 | -4.17800 | 3.15300 |
| H | 6.05200 | -5.94500 | 2.85000 |
| H | 5.86100 | -4.17900 | 2.72000 |
| H | 7.41600 | 1.29200 | 3.28300 |
| H | 5.85500 | 2.04000 | 2.85400 |
| H | 2.99000 | 5.61600 | 3.07500 |
| H | 3.71500 | 6.26500 | 1.60200 |
| H | -2.35700 | 7.33500 | 3.30100 |
| H | -3.20200 | 6.73900 | 1.85200 |
| H | -8.09300 | 2.51500 | 3.07300 |
| H | -7.11200 | 1.03800 | 2.90100 |

| | | | |
|---|---|---|---|
| C | -1.52600 | -7.08600 | 3.89600 |
| C | -1.19600 | -7.20500 | 2.41700 |
| H | -0.11100 | -7.29100 | 2.30200 |
| O | -2.50700 | -6.18500 | 4.13100 |
| H | -2.68900 | -6.22300 | 5.08300 |
| O | -1.01400 | -7.74100 | 4.76200 |
| H | -1.64900 | -8.13700 | 2.06300 |
| C | 6.62700 | -5.18300 | 0.95100 |
| O | 7.74600 | -4.43700 | 0.99800 |
| O | 6.40400 | -5.89600 | 0.01100 |
| H | 7.70000 | -3.79400 | 1.71700 |
| C | 7.38600 | 2.15800 | 1.33200 |
| O | 7.35400 | 3.49100 | 1.38200 |
| O | 7.97200 | 1.57700 | 0.45400 |
| H | 6.75900 | 3.83100 | 2.07800 |
| C | -5.42900 | -5.15900 | 1.78600 |
| O | -4.67400 | -6.26600 | 1.85800 |
| O | -6.35800 | -5.07500 | 1.02800 |
| H | -3.81100 | -6.07300 | 2.26700 |
| C | -8.27000 | 1.61900 | 1.15400 |
| O | -8.92000 | 0.44100 | 1.17900 |
| O | -8.40500 | 2.37600 | 0.23200 |
| H | -8.58200 | -0.12200 | 1.88700 |
| C | -2.00500 | 8.46700 | 1.50100 |
| O | -2.84900 | 9.45800 | 1.84400 |
| O | -1.16000 | 8.63700 | 0.66900 |
| H | -3.46600 | 9.17300 | 2.53100 |
| C | 5.12100 | 5.81600 | 3.17000 |
| O | 5.16200 | 7.03000 | 3.73300 |
| O | 6.03900 | 5.05100 | 3.30300 |
| H | 4.36200 | 7.53800 | 3.53900 |

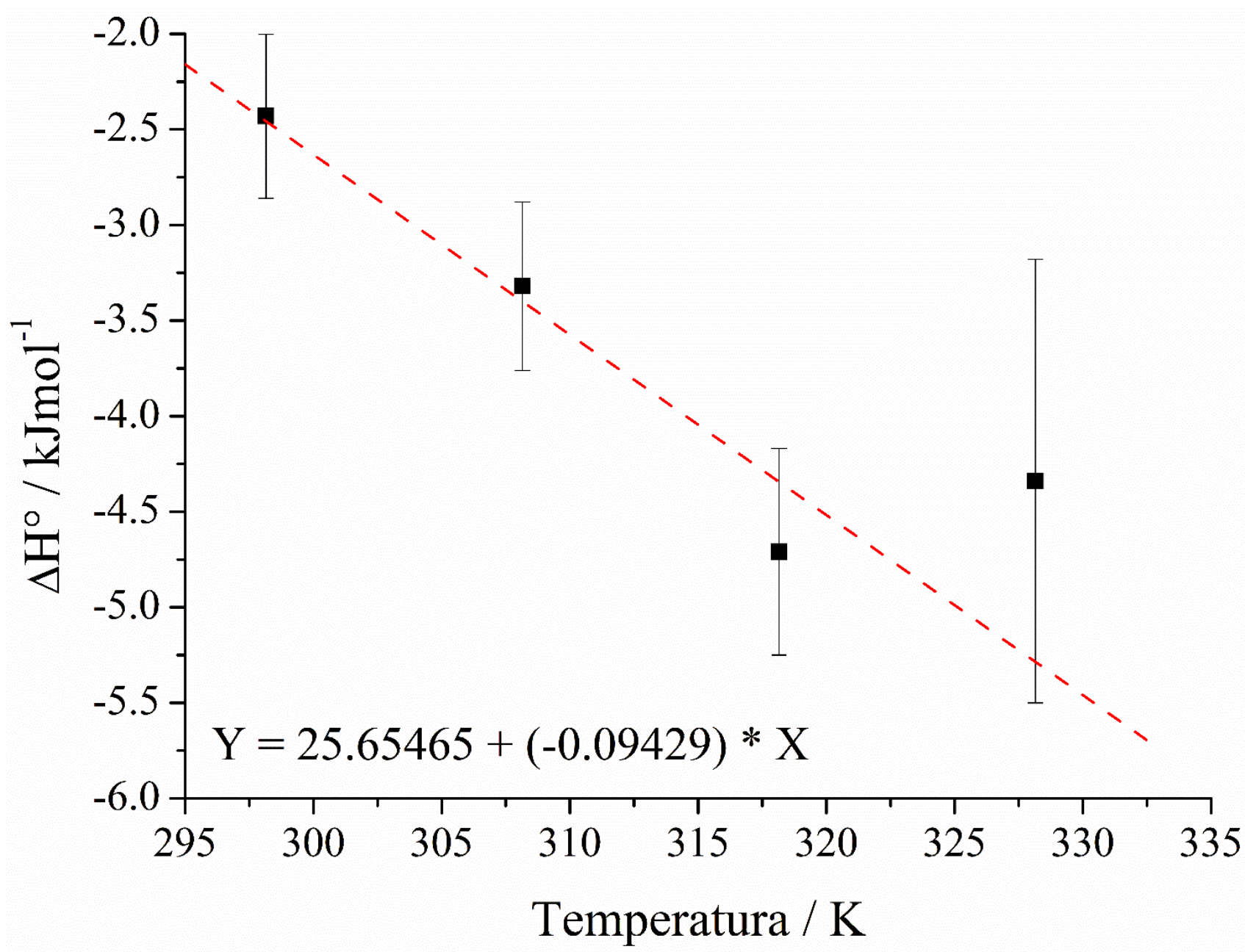


a)

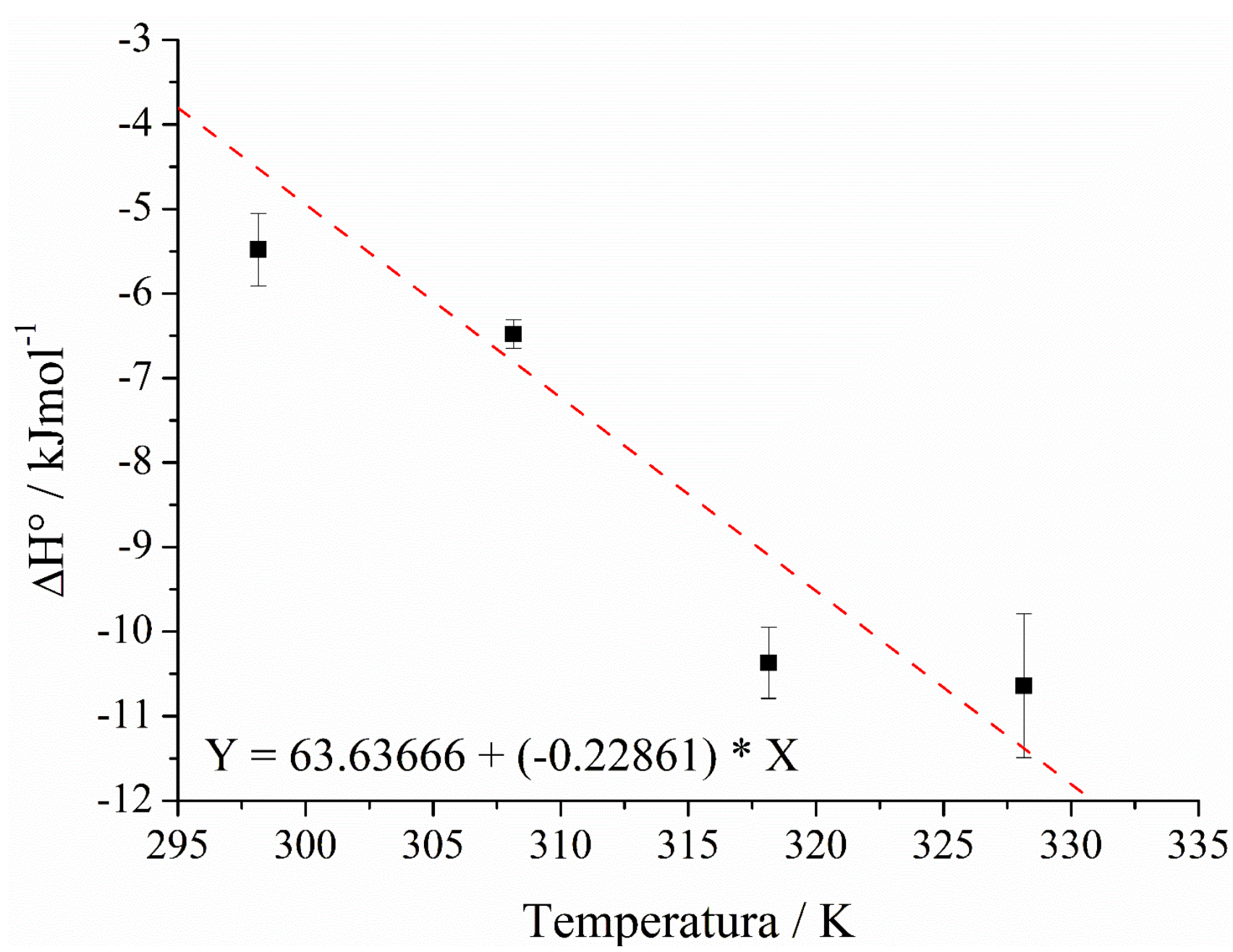


b)

Figure S3: heat capacity change for CM-βCD:SEL supramolecular systems: a) variable stoichiometry and b) fixed stoichiometry (n = 1).

Supporting Information (Table S2)

Table S2: $^{1}H$ and $^{13}C$ NMR (400 MHz) chemical shift for SEL in $D_2O$ with 5% of $DMSO_{d6}$ and the numbered chemical structure of SEL molecule.

| **Attribution** | | **δ $^{1}$H** | **δ $^{13}$C** |
|---|---|---|---|
| 1 | $-CH_2$ | 3.96 | 43.30 |
| 2 | -C | --- | 79.70 |
| 3 | -CH | 3.22 | 80.18 |
| 4 | --- | --- | --- |
| 5 | $-CH_2$ | 3.18 / 2.83 | 61.67 |
| 6 | -CH | 3.88 | 61.71 |
| 7 | $-CH_3$ | 1.24 | 12.52 |
| 8 | -C | --- | 135.66 |
| 9 | -CH | 7.38 | 129.39 |
| 10 | -CH | 7.45 | 129.07 |
| 11 | -CH | 7.41 | 127.51 |
| 12 | -CH | 7.45 | 129.07 |
| 13 | -CH | 7.38 | 129.39 |
| 14 | $-N-CH_3$ | 2.93 | 35.67 |

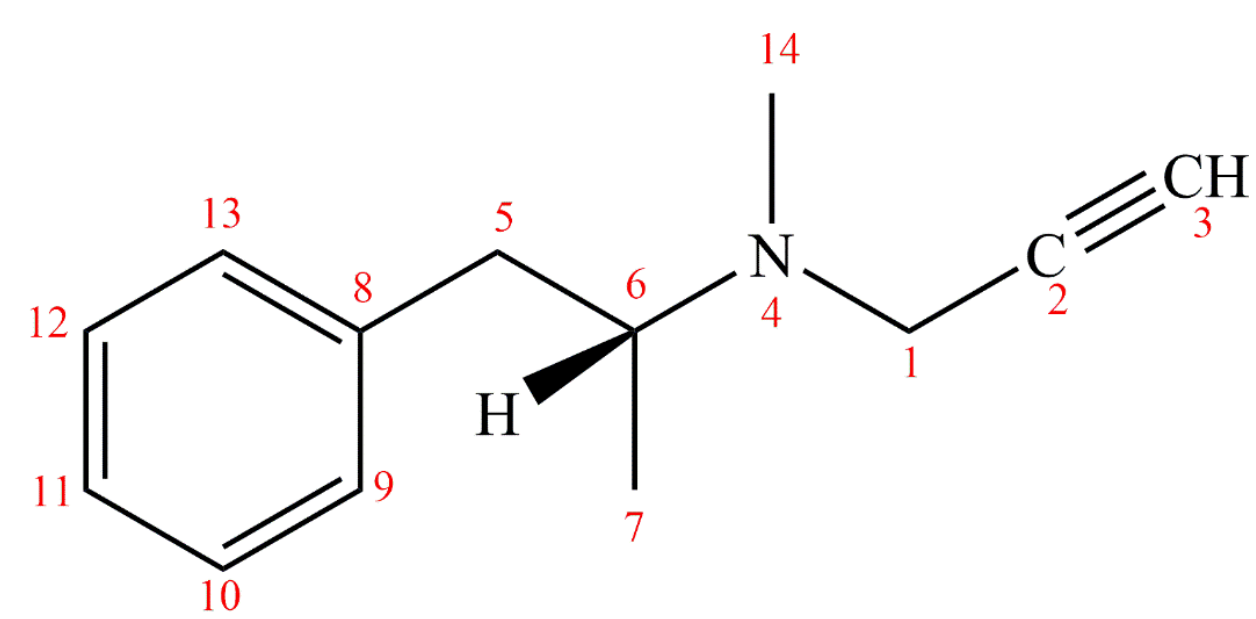

Supporting Information (Figure S4)

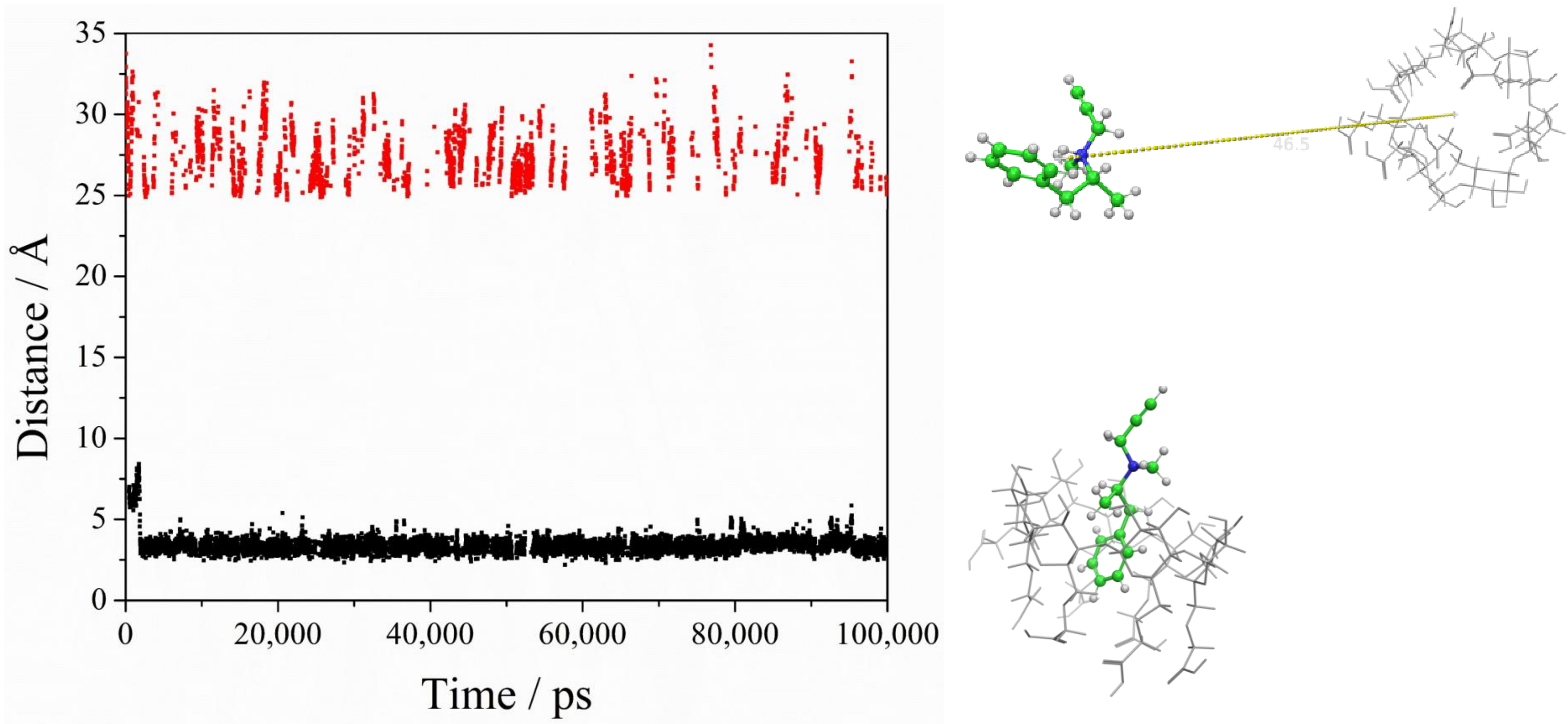


Figure S4: Calculated distances between the SEL center of mass and CM-βCD:SEL over the 1:1 MD simulation. Black dots for the inclusion complex and red dots for the non-inclusion complexes, both with the structural representation in the right side.

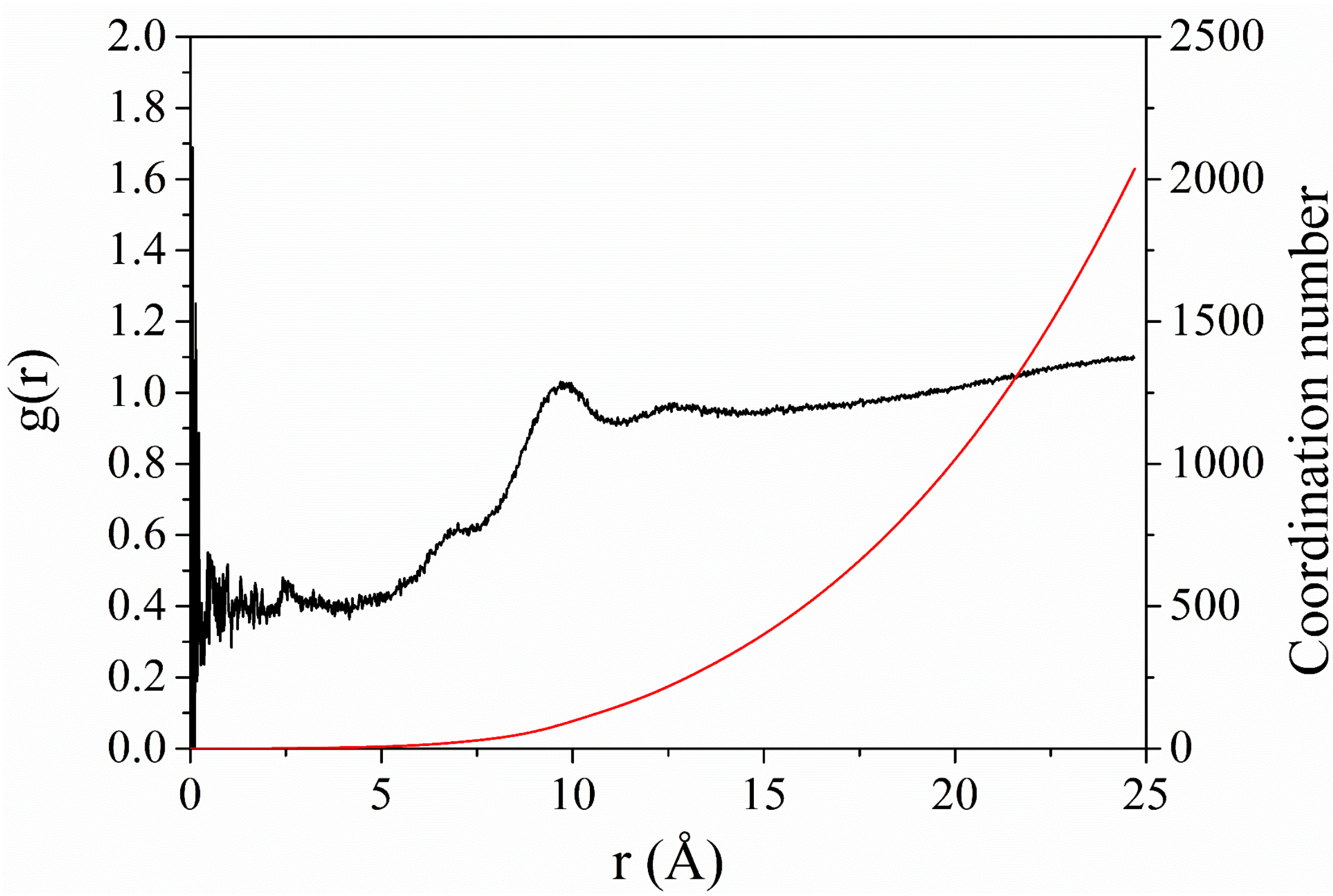


Figure S5: Radial distribution function of the center of mass of the complex and water solvent of the 1:1 stoichiometry.
